# Language Proficiency Modulates the Recruitment of Non-Classical Language Areas in Bilinguals

**DOI:** 10.1371/journal.pone.0018240

**Published:** 2011-03-24

**Authors:** Matthew K. Leonard, Christina Torres, Katherine E. Travis, Timothy T. Brown, Donald J. Hagler, Anders M. Dale, Jeffrey L. Elman, Eric Halgren

**Affiliations:** 1 Department of Cognitive Science, University of California San Diego, La Jolla, California, United States of America; 2 Multimodal Imaging Laboratory, University of California San Diego, La Jolla, California, United States of America; 3 Department of Radiology, University of California San Diego, La Jolla, California, United States of America; 4 Department of Neurosciences, University of California San Diego, La Jolla, California, United States of America; 5 Kavli Institute for Brain and Mind, University of California San Diego, La Jolla, California, United States of America; Cuban Neuroscience Center, Cuba

## Abstract

Bilingualism provides a unique opportunity for understanding the relative roles of proficiency and order of acquisition in determining how the brain represents language. In a previous study, we combined magnetoencephalography (MEG) and magnetic resonance imaging (MRI) to examine the spatiotemporal dynamics of word processing in a group of Spanish-English bilinguals who were more proficient in their native language. We found that from the earliest stages of lexical processing, words in the second language evoke greater activity in bilateral posterior visual regions, while activity to the native language is largely confined to classical left hemisphere fronto-temporal areas. In the present study, we sought to examine whether these effects relate to language proficiency or order of language acquisition by testing Spanish-English bilingual subjects who had become dominant in their second language. Additionally, we wanted to determine whether activity in bilateral visual regions was related to the presentation of written words in our previous study, so we presented subjects with both written and auditory words. We found greater activity for the less proficient native language in bilateral posterior visual regions for both the visual and auditory modalities, which started during the earliest word encoding stages and continued through lexico-semantic processing. In classical left fronto-temporal regions, the two languages evoked similar activity. Therefore, it is the lack of proficiency rather than secondary acquisition order that determines the recruitment of non-classical areas for word processing.

## Introduction

Bilingualism is a fascinating and complex phenomenon of culture, identity, and skill that deserves attention for its prominence among modern societies and also for what it can tell us about language and cognitive ability more broadly. Previous studies have shown that proficiency modulates lexico-semantic processing in both languages in bilinguals, as indexed by reaction time priming tasks [1]–[4], electroencephalographic methods [5], [6], and brain imaging studies [7]–[11]. The Revised Hierarchical Model (RHM) for bilingual language representation predicts these findings as arising from proficiency-modulated links between the first (L1) and second (L2) languages and a supramodal conceptual store [12].

It is unclear how these links are mediated in the neural systems that underlie word processing in the two languages and how they change when one becomes more proficient in the second-learned language. In a previous study, we combined magnetoencephalography (MEG) and structural MRI to show that when reading words in Spanish and English, native Spanish speakers who are still dominant in Spanish have overlapping activity for both languages in classical left fronto-temporal regions during lexico-semantic processing [13]. In contrast, activity to words in the less proficient English additionally involves right hemisphere and bilateral secondary visual regions such as lateral and ventral occipitotemporal cortex (LOT and VOT) as early as ∼135 ms, and continuing through long latency time windows (∼400 ms after a word was shown). Furthermore, only less familiar words in the less familiar language showed this pattern, suggesting that these regions may become active when the initial task of identifying words is more difficult [14], [15]. Several imaging studies have found more distributed activity for the less proficient language [8], [13], [16]–[20], however this is a controversial interpretation [7].

In the present study, we tested native Spanish speakers who had become dominant in English to examine whether greater activity in non-classical language areas is associated with lower proficiency (where Spanish would evoke greater activity in LOT and VOT) or order of acquisition (where English would evoke greater activity in these areas, identical to our previous study). We also sought to examine whether bilateral visual activity that occurs after sensory-perceptual processing is related to the visual paradigm we used in our previous study. Therefore, we presented subjects with words in both the visual and auditory modalities to confirm that bilateral visual activity in object processing regions like LOT is lexico-semantic in nature, and not tied to the stimulus modality. We found that although the pattern was weaker than in our previous study, a lack of proficiency rather than secondary acquisition order was clearly associated with activity outside of classical language areas, and that this effect occurred for words in both the visual and auditory modalities.

## Materials and Methods

### Ethics Statement

This study was approved by the Institutional Review Board at the University of California, San Diego. All subjects gave informed, written consent prior to enrolling in the study, and were paid for their time.

### Subjects

Sixteen healthy right-handed adults (nine females; age range  = 20–28 years; mean  = 22.31 years) participated in this study. Participants reported no history of psychological or neurological impairment, and all had completed at least some college. All were native Spanish speakers who began acquiring English as a second language early when they entered school (mean age of acquisition  = 5.27 years, SD  = 1.44 years). Language history and proficiency in both languages were assessed by a detailed questionnaire that asked subjects to rate on a scale from 1–10 their language abilities for speaking, understanding, and reading, and to indicate the sources/methods that contributed to learning each language (adapted from [21]). One subject's questionnaire was excluded due to improper data collection. No subjects indicated higher proficiency in Spanish for any of these domains, although three indicated equal abilities for speaking (Spanish mean  = 7.73, English mean  = 8.87), and two subjects were equally proficient in reading (Spanish mean  = 6.93, English mean  = 8.73). For understanding, seven subjects noted that they comprehended both languages equally (Spanish mean  = 8.27, English mean  = 8.93). Furthermore, subjects indicated a strong preference for how often they would choose to read in English (89.33% of the time), which was also the case for speaking (59.6% vs. 37.4% Spanish; three subjects knew a third language). Subjects also responded that they currently receive more English exposure in general (72.6% English vs. 29.4% Spanish), although their relative exposures during childhood were more balanced (55% English vs. 45% Spanish). From these self assessments, we concluded that all subjects in this study were more proficient and comfortable in their second learned language, English.

### Task

The task presented here is nearly identical to that in our previous study [13]. Subjects performed a semantic size judgment task to words (“Does this object fit into a shoebox?”) while MEG was recorded. The difference between this study and the previous one is that words were presented in both the visual and auditory modalities in separate blocks. The first block was visual words followed by a block of auditory words, each of which consisted of ten stimuli that repeated six times each in random order. These blocks were meant to provide practice and training, and to set up a repetition priming effect. They were not included in the analyses described below. The order of the next blocks (visual vs. auditory) was counterbalanced across subjects, and each block contained a mix of repeated stimuli (‘old’) from the practice blocks, and words that were presented one time only (‘new’). In the following two visual and two auditory blocks, subjects saw and heard 60 new words and six more repetitions of each of the 10 old words in each modality (**Figure S1**). No new or old words repeated across modalities or languages, and the order of the stimuli in each block was randomized with the constraint that there must be an average of 19 words (10 new and 9 old, or ∼45 seconds) between presentations of a given old word.

This task was designed to allow for comparisons between activity in the two languages. Of specific interest were earlier components related to modality-specific lexical encoding (at ∼170 ms to peak in the visual modality and ∼100 ms for auditory), as well as later components indexing lexico-semantic associations (peaking at ∼400 ms in both modalities). The later component, the N400, has been intensely studied with EEG where it is found to be modulated by the degree of difficulty of contextual integration, stimulus frequency, and stimulus repetition [22]. The N400m is the magnetic counterpart of the N400, with similar cognitive correlates, but is easier to localize. Both early and late components are generated by current flows within the apical dendrites of cortical pyramidal cells, with the earlier peak due to feedforward synaptic excitation, and later components due to more associative synaptic inputs [23]. We predicted that some areas would show N400 repetition suppression effects ∼400 ms after stimulus presentation in both modalities, and that the locations of some of these effects would differ between languages.

For each visual trial a written word was presented centrally for 300 ms, followed by a masking fixation cross for 2000–2200 ms, during which subjects made their size judgment responses by lifting their index fingers from a fiber optic response paddle (the response hand mappings were counterbalanced across subjects). The fixation cross was on the screen during the entire trial for auditory blocks. All words were concrete, highly imageable objects, and were both high frequency (Spanish mean occurrences per million  = 39.71, English occurrences per million  = 35.23, *p*>0.6) and early-learned words in each language of presentation. Some objects were easier to judge than others; while “bug” and “elephant” clearly do and do not fit into a shoebox, “apron” and “shirt” are less obvious. As the task was designed to activate word meanings implicitly, these differences do not affect our current analyses, however future studies may examine these variables parametrically. Visual words were equated for word length (Spanish mean  = 5.46 letters; English mean  = 5.61 letters). Auditory words were recorded in a soundproof booth with a condenser microphone by a fluent Spanish-English bilingual speaker who did not have a strong accent in either language. The stimuli were edited to be the shortest possible length while maintaining intelligibility (mean length Spanish  = 470 ms, SD  = 84 ms; mean length English  = 528 ms, SD  = 99 ms) and all stimuli were equated for mean intensity at 65 dB. Due to the semantic constraints and inherent phonemic differences between Spanish and English, it was not possible to equate the words in the frequency domain. No auditory stimuli were homophones either within or across languages.

All blocks in one language (both visual and auditory) were presented sequentially, followed by three blocks of non-verbal line drawings of objects, and then the six blocks of stimuli in the other language. Due to persistent differences in activity evoked by each modality, visual and auditory words were analyzed separately. The order of the languages was counterbalanced across participants. Although these subjects were highly proficient at code switching, we wanted to examine the relative organizations of the two languages, so it was necessary to minimize the effects of attentional and language switching mechanisms. Therefore, all interactions with the subjects and instructions for each block were presented in the language of the subsequent block by a fluently bilingual research assistant.

### MEG Recording

Subjects sat in a magnetically shielded room (IMEDCO-AG, Switzerland) with their heads in a Neuromag Vectorview helmet-shaped dewar containing 102 magnetometers and 204 planar gradiometers (Elekta AB, Helsinki, Finland). Data were collected at a continuous sampling rate of 2000 Hz with minimal filtering (0.1 to 200 Hz). The locations of four non-magnetic coils affixed to the subjects' heads were digitized along with the main fiduciary points (nasion and preauricular points) for subsequent coregistration with high-resolution structural MR images. Subjects were instructed to remain as still as possible during the ∼45 minute recording session, and head position indicator (HPI) measurements at the beginning of each stimulus block (approximately every 3–4 minutes) confirmed that the subjects moved minimally (average 8.82 mm Euclidean distance from the beginning to the end of the session). With the exception of one subject, movement in all directions was less than 1.7 cm. One subject moved 2.7 cm in the front-to-back direction, however the average head locations between runs for the two languages were less than 2.1 cm apart.

### Anatomically-constrained MEG analysis

The data were analyzed according to the same procedures described in our previous study [13]. Briefly, we used a multimodal imaging approach that constrains the MEG activity to the cortical surface as determined by high-resolution structural MRI [24], [25]. This noise-normalized linear inverse technique, known as dynamic statistical parametric mapping (dSPM) provides source estimates that can be visualized across time on the cortical surface as movies. EEG and MEG are not sufficient for unambiguous current source localization because any given extracranial electromagnetic field is consistent with an infinite number of possible equivalent current dipole (ECD) configurations in the brain. The dSPM method reduces this ambiguity with the reasonable assumption that sources are located in the cortex, and the source estimates in language tasks have been validated by comparison with direct intracranial recordings [26]–[29].

Noise normalized dSPMs were calculated for each subject and then averaged onto a common space as a group mean of the estimates. From the group mean time courses of the activity, temporal windows were selected for statistical analysis in various regions of interest (ROIs). Twelve ROIs were selected based on *a priori* hypotheses. These ROIs overlapped with the regions that were used in our previous study, however they were drawn based on a grand average across all subjects and all conditions in the new dataset, so they differed slightly in location and extent (**Figure S2**). The group average F-values (represented by the color bars in the figures below) from the time course of the mean activity within each ROI were entered into a repeated-measures analysis of variance (ANOVA) with language (Spanish vs. English) and repetition (new vs. old) as within-subject factors. All reported *p*-values are uncorrected for multiple comparisons.

## Results

### Reaction Time

Reaction times were measured from the onset of the stimulus to the time the subject lifted his or her finger from the response paddle. Reaction times for visual and auditory words were entered into separate repeated-measures ANOVAs, with language (Spanish vs. English) and repetition (new vs. old) as factors.

**Figure 1:**
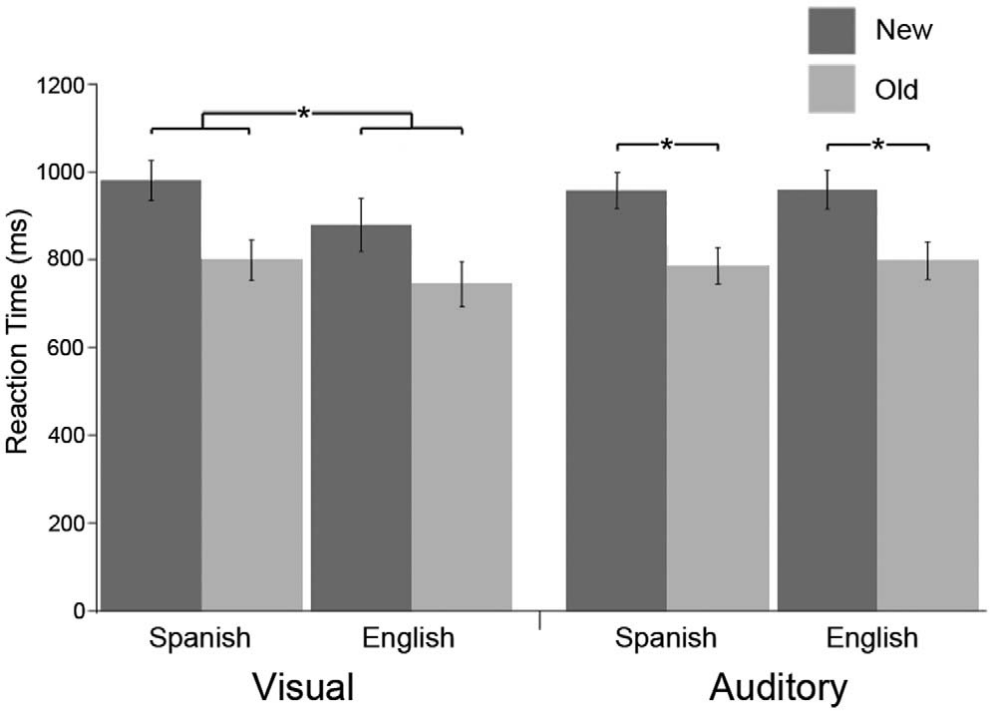
Mean reaction times for 16 subjects. Subjects responded faster to old words in both languages and modalities, and responded faster to English words in the visual modality. Error bars represent the standard error of the mean.

For visual words, subjects responded significantly faster to old words [F(1,15) = 143.66, *p*<0.0001], and also showed an effect of words in English being faster than words in Spanish [F(1,15) = 7.66, *p* = 0.014] (**Figure 1**). Additionally, there was a marginally significant interaction [F(1,15) = 4.35, *p* = 0.055] with new English words faster than new Spanish words [t(15) = 3.12, *p* = 0.007]. There was a marginal effect of English old words being faster than Spanish old words [t(15) = 1.97, *p* = 0.067].

In the auditory modality, there was only a main effect of repetition, with old words being faster than new words [F(1,15) = 185.70, *p*<0.0001] (**Figure 1**). There were no effects of language and the interaction was not significant.

### Early Visual Word Encoding (∼170 ms)

**Figure 2:**
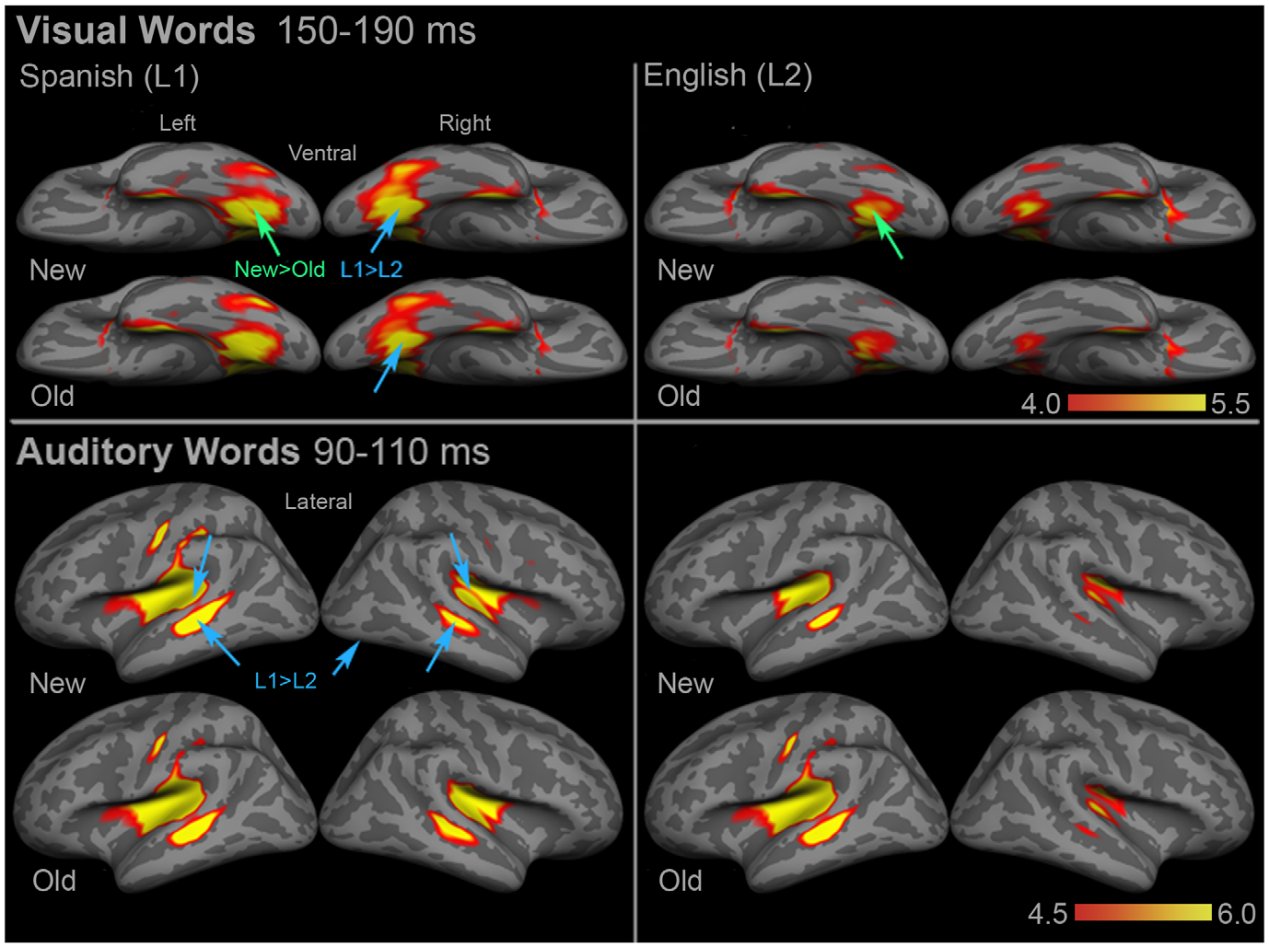
Group dSPM of the mean activity during early visual (top) and auditory (bottom) word encoding. In both modalities, activity appears to be strongly lateralized for English but largely bilateral for Spanish. For visual words, left VOT showed a marginal repetition effect (green arrows), and right VOT showed a trending Spanish>English language effect (blue arrows). For auditory words, right planum temporale, bilateral anterior STS, and right posterior fusiform showed Spanish>English effects (blue arrows). Left planum temporale showed a trending Spanish>English effect. See **Supplementary Figure S2** for ROI locations and names. Color bars represent square root of F values, which are a measure of signal-to-noise.

**Figure 3:**
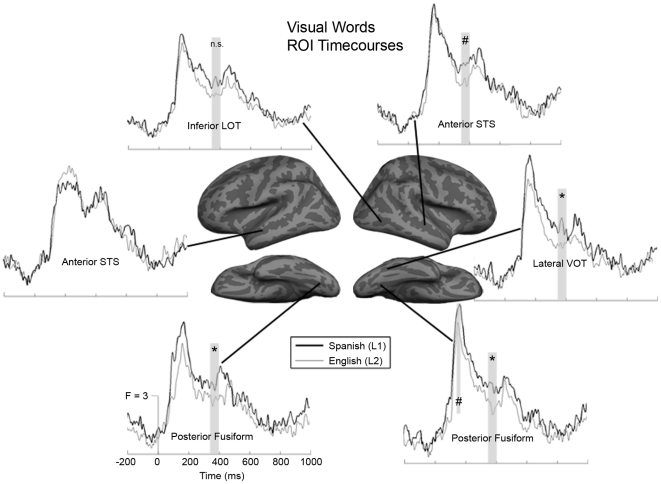
Average time courses for selected ROIs to new visual words. Several regions show significant (denoted by *) or marginal (denoted by #) Spanish (thick lines) > English (thin lines) effects during the early (150–190 ms) and late (350–400 ms) time windows (gray bars). Responses appear generally greater over an extended time period for Spanish than for English, especially in right hemisphere and posterior areas.

For visual words, the first peak in ventral occipitotemporal regions occurred ∼170 ms post-stimulus onset. The group mean dSPM from the posterior fusiform ROI was averaged across a 40 ms time window from 150–190 ms, and we compared the activity in the left and right regions across conditions (**Figure 2** **top and** **Figure 3**). In the left hemisphere, there was only a marginal effect of repetition with new>old, [F(1,15) = 4.17, *p* = 0.059]. In the right hemisphere, there was a trend toward a main effect of language, with Spanish>English [F(1,15) = 3.09, *p* = 0.099]. None of the interactions were significant.

### Early Auditory Word Encoding (∼100 ms)

**Figure 4:**
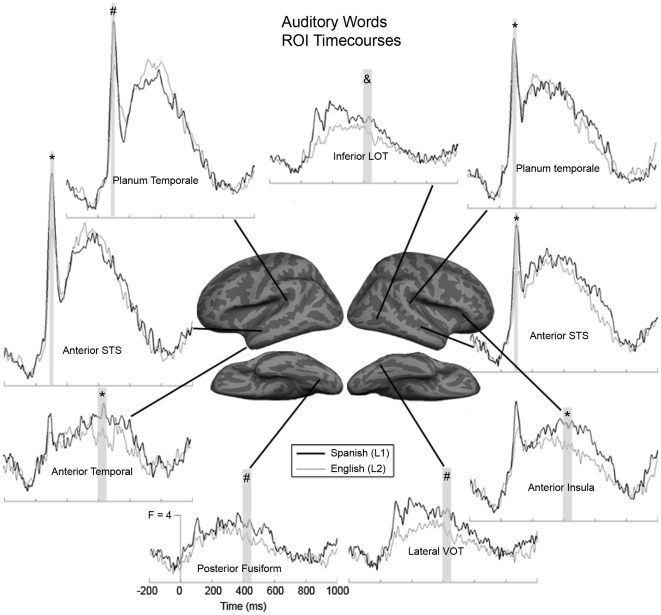
Average time courses for selected ROIs to new auditory words. Overall responses appear greater over an extended time period for Spanish (thick lines) than for English (thin lines), especially in right hemisphere and posterior areas. Planum temporale and anterior STS show significant (denoted by *) or marginal (denoted by #) Spanish>English effects during the early encoding stage (90–110 ms), while other areas show language effects during the late lexico-semantic stage (400–450 ms). Of particular interest is the activity evoked by auditory words in right inferior LOT and lateral VOT, which are typically associated with visual object processing. The right inferior LOT area also has a significant new>old effect in Spanish, but not in English (denoted by &).

For auditory words, the first major peak in bilateral superior temporal regions occurred ∼100 ms post-stimulus onset. The group mean dSPMs were averaged across a 20 ms time window from 90–110 ms, and we compared the activity across conditions in the bilateral temporal regions where the peak was maximal (**Figure 2** **bottom and** **Figure 4**). In left planum temporale, there was a trend toward a main effect of language, with Spanish>English [F(1,15) = 3.47, *p* = 0.082]. In the right hemisphere homologue, there was a significant main effect of language in the same direction, [F(1,15) = 4.72, *p* = 0.046]. Anterior superior temporal sulcus (STS) also showed a strong peak at ∼100 ms, and both left ([F(1,15) = 11.55, *p* = 0.004]) and right ([F(1,15) = 14.78, *p* = 0.002]) regions showed significant main effects of language in the Spanish>English direction. Finally, right posterior fusiform showed a main effect of language with Spanish>English, [F(1,15) = 4.47, *p* = 0.052].

### Lexico-semantic Responses to Visual Words (∼400 ms)

**Figure 5:**
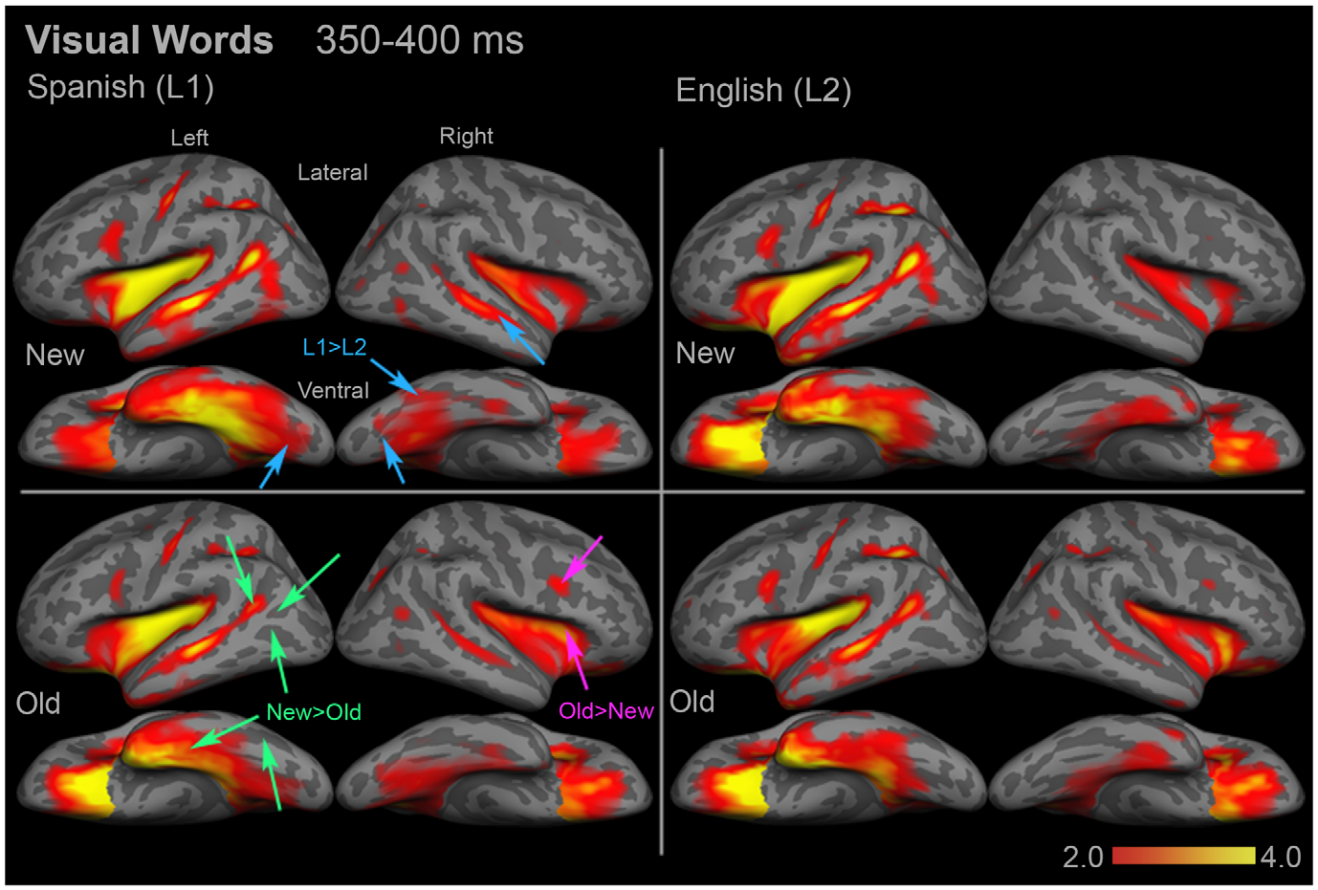
Group dSPM images of the mean activity evoked by visual words from 350–400 ms. Several regions in bilateral posterior and right anterior temporal cortex showed significant Spanish>English effects (blue arrows). The activity associated with the N400m in classical left temporal and frontal language areas was not significantly different between Spanish and English. Stimulus repetition effects (green arrows) were also significant in many regions, including two regions with old>new effects (magenta arrows). See **Supplementary Figure S2** for ROI locations and names. Color bars represent square root of F values, which are a measure of signal-to-noise.

As in our previous study, there were several regions in both hemispheres that showed significant activity to words in both languages, which peaked around 400 ms. During a 50 ms time window from 350–400 ms, multiple regions showed significant new>old repetition effects that are characteristic of N400 modulation. In the left hemisphere, inferior temporal cortex ([F(1,15) = 6.92, *p* = 0.019]), inferior LOT ([F(1,15) = 8.21, *p* = 0.012]), superior LOT ([F(1,15) = 5.10, *p* = 0.039]), and posterior STS ([F(1,15) = 15.48, *p* = 0.001]) had significantly greater responses to new words (**Figure 5**). Lateral VOT also showed a trend toward a significant main effect of repetition, [F(1,15) = 3.27, *p* = 0.091]. In the right hemisphere, anterior insula ([F(1,15) = 6.39, *p* = 0.023]) and the inferior pre-central sulcus ([F(1,15) = 6.49, *p* = 0.022]) showed significant repetition effects, however they were both old>new.

There were several areas that showed significant Spanish>English language effects (**Figure 3** **and** **Figure 5**). In the left hemisphere, only posterior fusiform cortex showed this pattern, [F(1,15) = 4.54, *p* = 0.05]. Left orbitofrontal cortex demonstrated a marginal interaction ([F(1,15) = 4.11, *p* = 0.061]), however this was driven by an old>new effect in Spanish ([t(15) = −2.05, *p* = 0.059]). In the right hemisphere, there was a significant Spanish>English effect in posterior fusiform cortex [F(1,15) = 5.24, *p* = 0.037]. There was also a non-significant trend toward a language effect in lateral VOT ([F(1,15) = 2.85, *p* = 0.112]), which was driven by a Spanish>English effect for new words, [t(15) = 2.21, *p* = 0.043]. A non-significant trend toward an interaction was also found in anterior STS ([F(1,15) = 2.82, *p* = 0.114]), which was driven primarily by a difference between new words, [t(15) = 2.04, *p* = 0.059].

### Lexico-semantic Responses to Auditory Words (∼400 ms)

**Figure 6:**
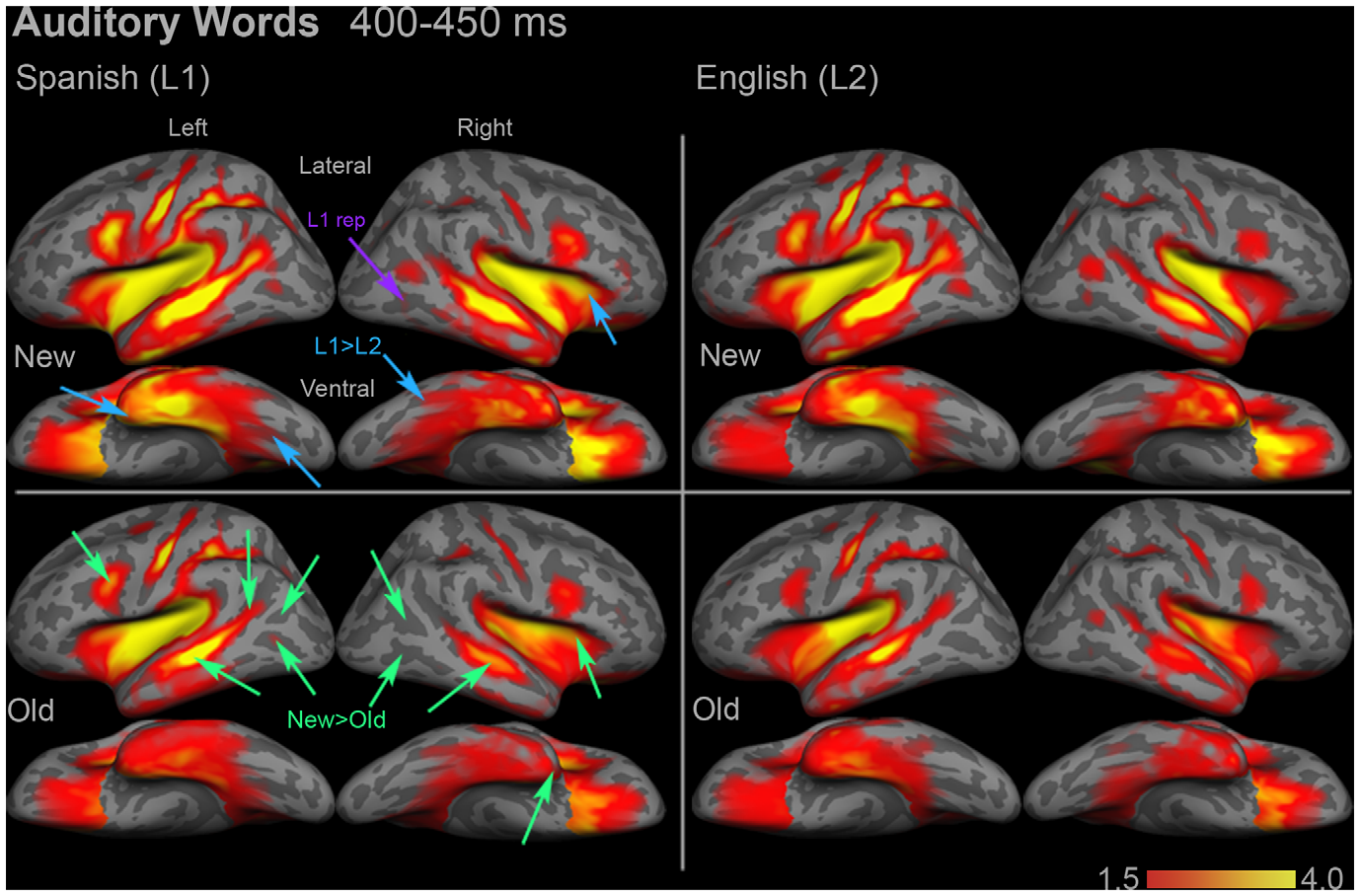
Group dSPM images of the mean activity evoked by auditory words from 400–450 ms. Several regions in both hemispheres showed Spanish>English effects (blue arrows). Note that the activity associated with the N400m in the regions of Wernicke's and Broca's areas was not significantly different between Spanish and English. Stimulus repetition effects (green arrows) were significant in many regions. One area, right inferior LOT, showed a new>old N400 effect in Spanish but not in English (purple arrow). See **Supplementary Figure S2** for ROI locations and names. Color bars represent square root of F values, which are a measure of signal-to-noise.

We selected a 50 ms time window from 400–450 ms that encompassed the largest between-condition differences for auditory words. During this time window, multiple regions showed significant repetition effects. In the left hemisphere, the inferior pre-central sulcus ([F(1,15) = 6.43, *p* = 0.023]), superior LOT ([F(1,15) = 12.65, *p* = 0.003]), anterior STS ([F(1,15) = 30.21, *p*<0.0001]), and posterior STS ([F(1,15) = 16.36, *p* = 0.001]) all showed significant new>old effects (**Figure 6**). Inferior LOT showed a marginal effect in the same direction, [F(1,15) = 3.90, *p* = 0.067]. In the right hemisphere, the following regions showed significant or trending new>old effects: anterior insula ([F(1,15) = 3.98, *p* = 0.065]), anterior temporal ([F(1,15) = 3.76, *p* = 0.071]), inferior LOT ([F(1,15) = 15.57, *p* = 0.001]), superior LOT ([F(1,15) = 11.50, *p* = 0.004]), and anterior STS ([F(1,15) = 6.21, *p* = 0.025]).

Several regions showed significant or marginal Spanish>English effects, including left anterior temporal ([F(1,15) = 5.08, *p* = 0.04]) and left posterior fusiform, [F(1,15) = 3.92, *p* = 0.066] (**Figure 4** **and** **Figure 6**). Also on the left, anterior STS showed a trend toward an interaction ([F(1,15) = 3.08, *p* = 0.10]), which was driven primarily by a strong repetition effect in English ([t(15) = 5.29, *p*<0.0001]).

In the right hemisphere, anterior insula showed a strong Spanish>English effect [F(1,15) = 14.61, *p* = 0.002]. Lateral VOT showed a trend toward an interaction between language and repetition ([F(1,15) = 3.37, *p* = 0.087]), which was driven by a trending difference between new words in each language, [t(15) = 1.89, *p* = 0.079]. Finally, inferior LOT showed a trend toward a significant interaction, [F(1,15) = 3.23, *p* = 0.092]. This was driven by a strong repetition effect in Spanish ([t(15) = 3.46, *p* = 0.003]) that was not present for English words.

## Discussion

We examined how language proficiency affects the recruitment of classical and other language areas during various stages of word processing in both the visual and auditory modalities. We used a multimodal imaging technique that combines the temporal resolution of MEG with the spatial resolution of MRI to distinguish activity in different brain regions during both early encoding (∼170 ms for visual words and ∼100 ms for auditory words) and late lexico-semantic (∼400 ms) processing stages. In this group of native Spanish speakers who began acquiring English around age six, and who have since become more proficient in English, responses to the less proficient Spanish were greater in multiple brain regions across both hemispheres beginning at the earliest stages of word encoding, and regardless of modality. This effect, though weak in some regions, persisted through ∼400 ms in both modalities, when lexico-semantic processing is thought to occur. During this time period bilateral occipito-temporal areas including posterior fusiform, lateral VOT, and LOT showed Spanish>English effects (or they showed new>old N400 effects in Spanish but not in English). Other right hemisphere regions including anterior STS and anterior insula showed similar effects, while no areas showed significant English>Spanish patterns. As in other studies using the same tasks with monolinguals, the most prominent activity during the N400 time window was estimated to lie in or near the classical language areas of the left hemisphere [30]. However, with the exception of the left temporal pole for auditory words, activity in these fronto-temporal areas did not differ significantly between English and Spanish.

Previously, we showed that in a group of Spanish-English bilinguals who were still dominant in their native language, the less proficient English recruited many of these same areas when subjects performed the visual task presented here [13]. However, it was unclear from that study whether proficiency or order of acquisition determined the extent of bilateral activity in English, and whether such activity is specific to the rather unnatural act of reading [31]. In the context of these findings, the present results suggest that regardless of modality, proficiency is the main factor in the recruitment of areas such as VOT and LOT during early encoding and late lexico-semantic processing stages, although other factors may contribute as well.

As one gains greater control over a language, both performance and the underlying neural substrates change to reflect increased proficiency, and presumably, more automatic processing [2], [4], [8], [10], [32]–[34]. We manipulated automaticity by inducing a repetition priming effect, in which some stimuli occurred once while others repeated multiple times over a delayed period. Particularly during the N400 time window, most of the between-language differences occurred between ‘new’ words, suggesting that the subjects' relative familiarity with words in each language influence the regions that are recruited to process them. Furthermore, the fact that regions such as right LOT showed significant new>old effects in the less proficient Spanish, but not in English, indicates that this region is performing a process that is modulated by language proficiency.

Our finding that order of acquisition is less relevant than proficiency in determining the amount of right hemisphere and posterior activity during early encoding and especially late semantic processing stages is important because it shows that models such as Kroll and colleagues' RHM [12] must include mechanisms to account for changes in language dominance. Behaviorally, when the second-learned language is the dominant language, it shows a pattern of cross-language priming effects that is similar to when the native language is dominant [34]. Neurophysiological and neuroimaging data support the idea that proficiency is crucial for determining the neural mechanisms recruited for each language, regardless of order of acquisition [5], [8]. Therefore, the notion of L1 and L2 as first and second languages must be qualified in relation to proficiency, which is a common issue for bilinguals in the United States, particularly those who are second or third generation Americans going to school in English, and who eventually become dominant in their second-learned language.

Our interpretation relies on previous work to conclude that proficiency drives the recruitment of non-classical language areas, yet there are some interesting differences between the English-dominant subjects and the Spanish-dominant group from our previous study. The magnitude of the between-language differences is smaller in the present study, and some regions did not show effects that appeared in our earlier work. We also did not replicate exactly the early visual word encoding effect, in which the right hemisphere fusiform region was only active for new words in English. There are several possible reasons for these discrepancies. It is possible that although proficiency is the main factor, order of acquisition interacts such that the native language retains much of its representational structure in the brain despite being used less frequently than the second-learned language (and it may even influence second-language representations [35]). Furthermore, proficiency is a somewhat poorly defined construct that is not independent of other factors such as daily use and age of acquisition. Age of acquisition is known to have strong effects on representations [36], [37], though it appears to affect different linguistic constructs than proficiency, including phonology, morphology, and syntax [20], [37], [38]. In contrast, proficiency has more profound effects on lexical and semantic processing, which are the focus of the present study. However, age and context of acquisition of individual words also play a role in how proficient one is at processing those particular words [37], [39]. For example, many of the concrete nouns in the present study were more likely to have been learned in a Spanish home context (“table”, “strawberry”, etc), compared to words that were learned in a school or work context (“giraffe”, “magnet”, etc) where English is the predominant language. Some of these words may also have been learned slightly earlier in one language or another. Future studies will examine the effects of acquisition context on neural representations to further refine the concept of proficiency.

It is also possible that the relative language proficiencies were different between the two groups. If subjects in the present study were more balanced, the magnitude of between-language effects should be weaker as both languages rely more exclusively on the classical language networks. Because we used self assessments (which are mostly designed to measure global dominance rather than precise levels of proficiency), it is difficult to determine whether this is the case. Future studies will employ objective measures of vocabulary knowledge to be able to correlate proficiency scores in each language with brain activity.

It is of great interest what differential recruitment of brain regions means in terms of the underlying processing mechanisms. It may be the case that even when there is greater involvement of right hemisphere resources, the mechanisms are the same as those in the classical left hemisphere language areas [40]. Our results may be consistent with this theory, and in fact help refine it. Since we have found significant overlap in the areas associated with word processing in both languages, it is clear that the neural substrate is at least partially shared. Any areas that differ (showing a less proficient>more proficient or a new>old pattern in one language but not the other) may be performing the same functions as the shared regions, which is supported by the presence of N400-like repetition effects in bilateral secondary visual areas. This would suggest that lower proficiency is a matter of recruiting more resources to process words.

An alternative hypothesis is that these supplementary regions are functionally distinct from the shared left fronto-temporal network. We suggested previously that the lexico-semantic repetition effects seen in secondary visual regions during bilingual language processing may be related to a more perceptual semantic system, compared to the abstract system that is mediated by classical language regions [13]. Presently, there is only indirect evidence from child language acquisition studies that supports this hypothesis [41]–[46], and further work is necessary to elucidate the functions of these regions during language processing. Whether these regions are performing similar or different functions as the classical left fronto-temporal network, their involvement in word processing in the less proficient language suggests that they could be neural markers of inexperience. Studies examining the neural substrates of learning and skill acquisition should take note of these regions and how their activity changes as skill increases.

While there are some differences between responses to visual and auditory stimuli that are likely due to inherent properties of the stimulus signal (visual being more ephemeral than auditory), we have also shown that especially during high-level language processing, modality does not greatly affect the pattern of representations in each language. In monolinguals, written and auditory words evoke activity in the same left fronto-temporal network during lexico-semantic processing [30], however it was previously unknown whether this was also true across languages in bilinguals. In addition to left fronto-temporal regions that show this supramodal response, supplementary regions that become active in the less proficient language such as LOT and VOT show a similar response across modalities. This suggests that the activity is not sensory or perceptual, but rather higher level and perhaps reflects similar lexico-semantic functions as the fronto-temporal networks.

Finally, our previous work suggested the existence of a right hemisphere analogue to the so-called “visual word form area” [47] that is selectively active in the less proficient language, or in any task in which reading words is more difficult [14], [15], [48], [49]. Although right posterior fusiform did not show the same repetition modulation in the English-dominant group (perhaps due to an interaction between proficiency and order of acquisition for reading), the trending Spanish>English effect in that region suggests a similar function. Furthermore, the analogous effect for auditory words in superior temporal regions suggests that this early encoding stage is affected by language proficiency in a similar manner as lexico-semantic processing. Given the early latency and location of this activity, it is possible that superior temporal regions function as an “auditory word form area”, which extracts lexical information from auditory stimuli and passes that information on to lexico-semantic regions, similar to the visual analogue. The existence of an auditory word form area has been a controversial topic [50], however this may be due to a lack of appropriate control stimuli that match the sensory characteristics of words across the spectrum over time. Our data support the notion of an area that provides a first-pass identification for words in the auditory modality, much like the posterior fusiform does for visual words.

The average person speaks or hears thousands of words per day. Our subjects had experienced tens of millions of words in each language, and for an average of 17 years, those languages were intermingled. Thus, bilingualism provides a powerful tool for studying how rich and complicated symbolic-semantic systems can be represented in the brain after extended, intense learning. Proficiency seems to drive many of the neural differences that occur between languages for single words, but it remains unknown whether non-classical language areas are recruited to a greater extent for later learned languages (or even later learned words), or for sentence and discourse level processing in the less proficient language. These questions provide a fascinating and fruitful platform for future study, which can help inform how experience and familiarity modulate neural representations.

## Supporting Information

Figure S1 **Task diagram.** Language order and modality order within language were counterbalanced across subjects.
(TIF)

Figure S2 **Regions of interest (ROIs) selected for statistical analysis.** Abbreviations: STS: superior temporal sulcus; LOT: lateral occipitotemporal; VOT: ventral occipitotemporal.
(TIF)
