# Supplementary figures and images for: Language Proficiency Modulates the Recruitment of Non-Classical Language Areas in Bilinguals
Source: PLoS One. 2011 Mar 24;6(3):e18240. doi: 10.1371/journal.pone.0018240 (PMC3063800; doi:10.1371/journal.pone.0018240)

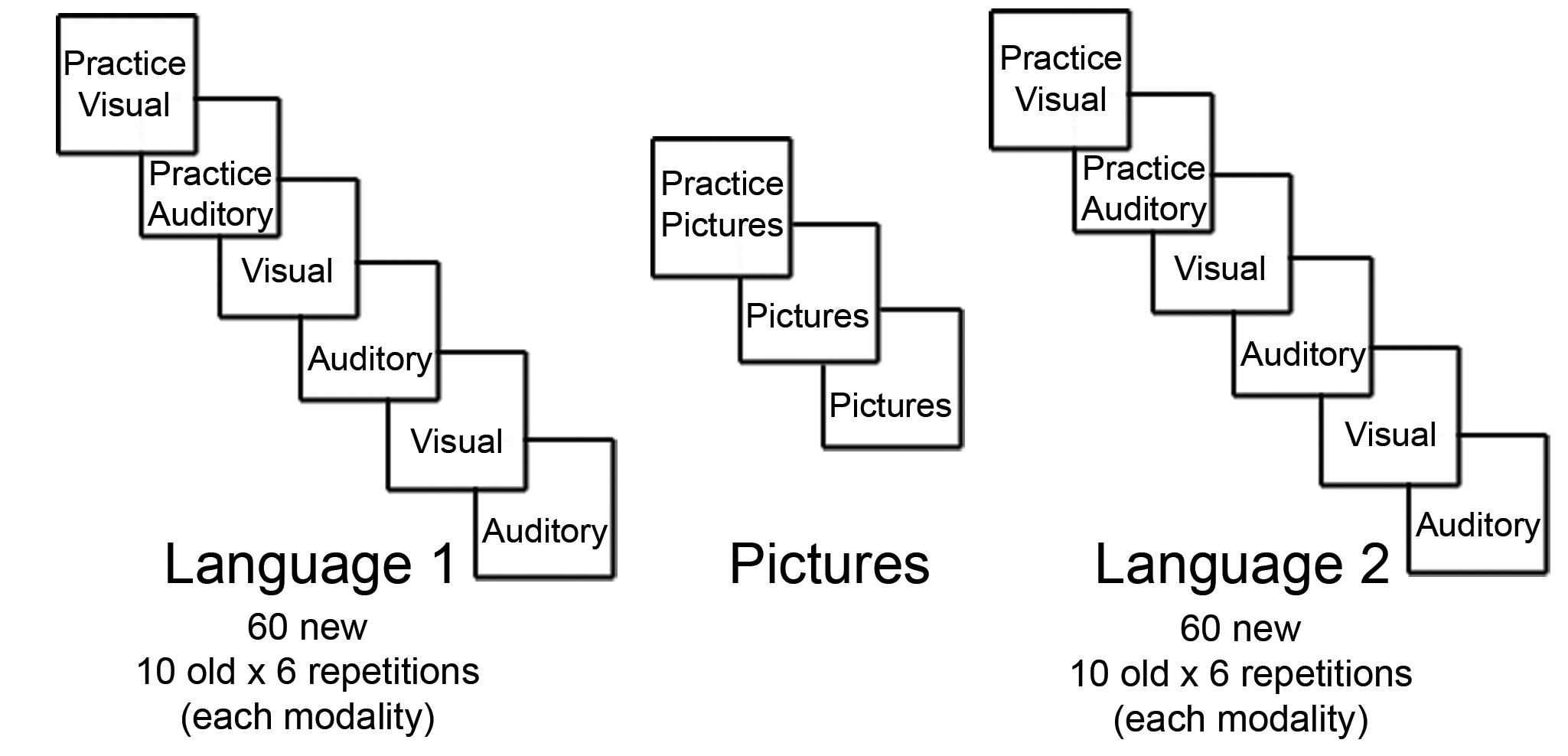

Supplement: Figure S1 — Task diagram. Language order and modality order within language were counterbalanced across subjects. (TIF) [file pone.0018240.s001.tif]

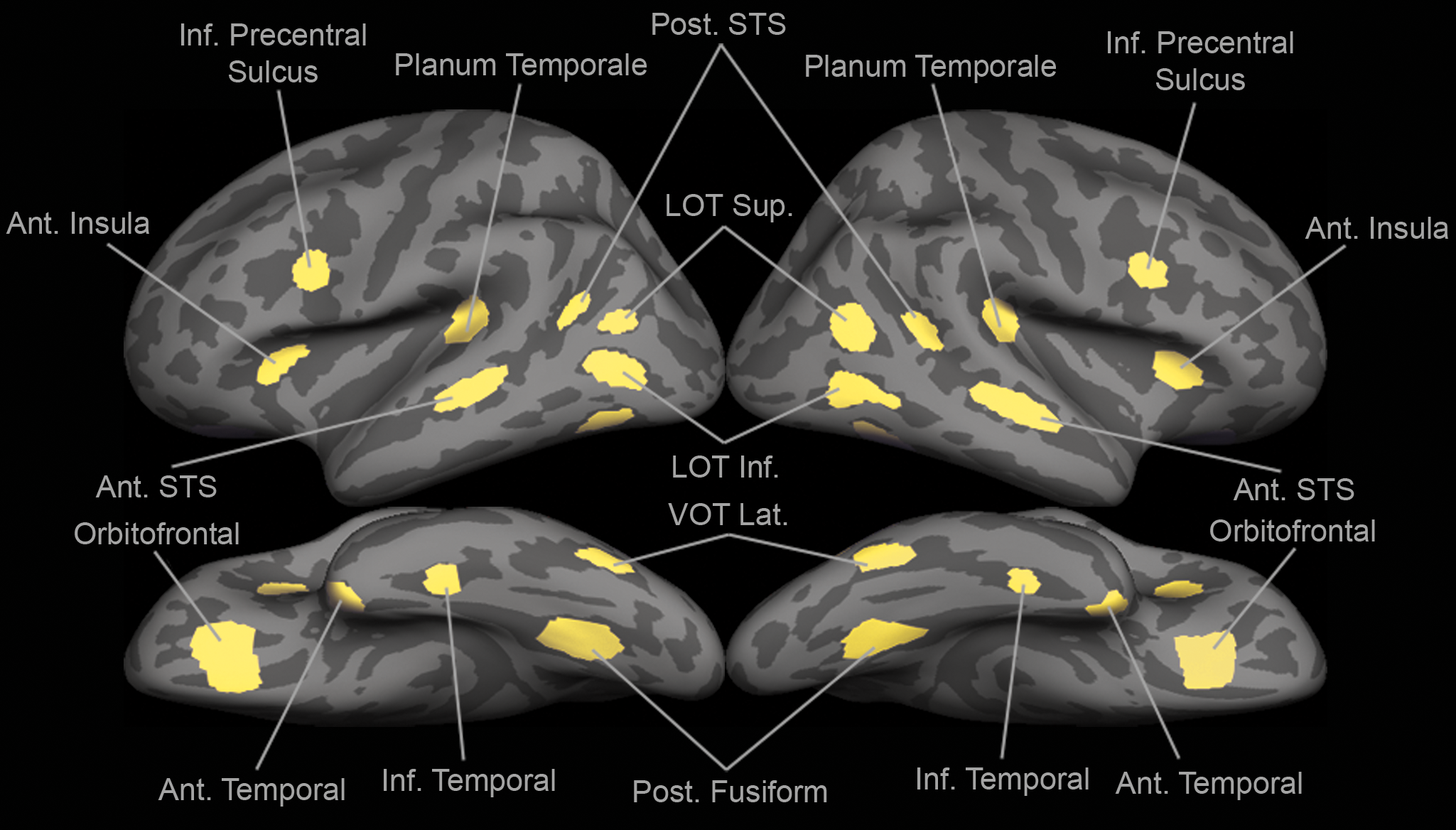

Supplement: Figure S2 — Regions of interest (ROIs) selected for statistical analysis. Abbreviations: STS: superior temporal sulcus; LOT: lateral occipitotemporal; VOT: ventral occipitotemporal. (TIF) [file pone.0018240.s002.tif]
